# Molecular epidemiology and antimicrobial resistance of extended-spectrum beta-lactamases-producing *Enterobacter cloacae* complex among mothers, neonates, healthcare workers and hospital environments in Tanga, Tanzania

**DOI:** 10.1093/jacamr/dlag161

**Published:** 2026-08-03

**Authors:** Athanas D Mhina, Neyaz A Khan, Ricardo Strauss, Dominik Schneider, Luisa Berckenhagen, Hagen Frickmann, Joyce R Mbwana, Daniel H Chercos, Anna Jaeger, Anangisye Malabeja, Mohamed Saleh, Simone Scheithauer, Ralf Krumkamp, Jürgen May, Mercy Chiduo, John P A Lusingu, Denise Dekker

**Affiliations:** National Institute for Medical Research, Tanga Centre, Tanga, Tanzania; One Health Bacteriology Group, Bernhard Nocht Institute for Tropical Medicine, Hamburg, Germany; Infectious Disease Epidemiology Department, Bernhard Nocht Institute for Tropical Medicine, Hamburg, Germany; Department of Infection Control and Infectious Diseases, University Medical Centre, Goettingen, Germany; Infectious Disease Epidemiology Department, Bernhard Nocht Institute for Tropical Medicine, Hamburg, Germany; Department of Microbiology and Hospital Hygiene, Bundeswehr Hospital, Hamburg, Germany; Institute for Microbiology, Virology and Hygiene, University Medicine Rostock, Rostock, Germany; National Institute for Medical Research, Tanga Centre, Tanga, Tanzania; One Health Bacteriology Group, Bernhard Nocht Institute for Tropical Medicine, Hamburg, Germany; Infectious Disease Epidemiology Department, Bernhard Nocht Institute for Tropical Medicine, Hamburg, Germany; National Institute for Medical Research, Tanga Centre, Tanga, Tanzania; Paediatric Department, Tanga Regional Referral Hospital, Tanga, Tanzania; Department of Infection Control and Infectious Diseases, University Medical Centre, Goettingen, Germany; Infectious Disease Epidemiology Department, Bernhard Nocht Institute for Tropical Medicine, Hamburg, Germany; German Centre for Infection Research, Hamburg-Lübeck-Borstel-Riems, Germany; Infectious Disease Epidemiology Department, Bernhard Nocht Institute for Tropical Medicine, Hamburg, Germany; German Centre for Infection Research, Hamburg-Lübeck-Borstel-Riems, Germany; Department of Medicine, University Medical Centre Hamburg-Eppendorf, Hamburg, Germany; National Institute for Medical Research, Tanga Centre, Tanga, Tanzania; National Institute for Medical Research, Tanga Centre, Tanga, Tanzania; One Health Bacteriology Group, Bernhard Nocht Institute for Tropical Medicine, Hamburg, Germany

## Abstract

**Background:**

The *Enterobacter cloacae* complex (ECC) is an emerging healthcare-associated pathogen and an increasing cause of infections in neonatal intensive care units. Its clinical relevance is driven by the rapid acquisition of multidrug resistance (MDR), particularly to extended-spectrum β-lactams (ESBLs) and carbapenems. This study investigated the distribution patterns and genomic diversity of MDR ECC in two hospitals in Tanzania.

**Methods:**

Between April 2022 and March 2023, samples from neonates, mothers, healthcare workers and hospital environments were analysed. ECC isolates underwent European Committee on Antimicrobial Susceptibility Testing–guided antimicrobial susceptibility testing and whole-genome sequencing to determine sequence types (STs), resistance determinants, plasmids and phylogenetic relationships.

**Results:**

A total of 132 ESBL-producing ECC isolates were recovered, with the majority originating from environmental sources (65.9%, *n*/*N* = 87/132). Neonatal colonization after ≥48 h of hospitalization was 10.2% (*n*/*N* = 18/176). Overall, 74.2% (*n*/*N* = 98/132) of isolates were MDR, while 10.6% (*n*/*N* = 14/132) showed resistance to carbapenems. Whole-genome sequencing of 120 isolates revealed predominance of *Enterobacter hormaechei* (93.3%, *n*/*N* = 112/120) and widespread ESBL genes, mainly *bla*_CTX-M-15_ (87.5%, *n*/*N* = 105/120). Carbapenemase genes *bla*_NDM-1_ and *bla*_NDM-5_ were identified in 10% (*n*/*N* = 12/120), frequently associated with IncX3 plasmids, while the colistin resistance gene *mcr*-10.1 was chromosomal. Forty-one distinct STs were detected, including 12 novel STs. ST346 was the most prevalent lineage. Repeated temporal clusters of identical STs, including ST346, were detected across environmental and human isolates.

**Conclusions:**

These findings demonstrate substantial MDR ECC colonization in neonatal wards, suggesting hospital environments as reservoirs for resistant ECC. Temporally clustered isolates across environmental and patient samples underscore the need for strengthened infection prevention and genomic surveillance.

## Introduction

*Enterobacter cloacae* complex (ECC) comprises several closely related *Enterobacter* species, with *Enterobacter hormaechei* being the most prevalent in the clinical setting.^1^ ECC are common gut colonizers but have emerged as important causes of multidrug-resistant (MDR) healthcare-associated infections worldwide.^2–4^ ECC pose a therapeutic challenge due to intrinsic resistance mediated by inducible chromosomal AmpC beta-lactamases, and the acquisition of extended-spectrum beta-lactamase (ESBL) genes, particularly *bla*_CTX-M*-*15_, further limits the use of third-generation cephalosporins.^5,6^ Treatment options are further restricted by rising resistance to carbapenems,^7^ polymyxins mediated by mobile colistin resistance (*mcr*) genes^8^ and fluoroquinolones.^9^ In addition to antimicrobial resistance (AMR), ECC harbour virulence-associated genes involved in adhesion, iron acquisition and environmental persistence, facilitating survival in hospital environments.^10,11^

MDR Enterobacterales, including ECC, are associated with high morbidity and mortality, particularly among neonates and other vulnerable hospitalized populations.^12–14^ In sub-Saharan Africa (SSA), increasing detection of MDR ECC in neonatal units is alarming, given the region’s limited infection prevention and control (IPC) measures.^15,16^ Colonization plays a critical role in the transmission and subsequent infections, with hospital environments and healthcare workers (HCWs) acting as important reservoirs.^17,18^

Molecular epidemiological studies have revealed substantial ECC diversity, with sequence types (STs) such as ST171, ST114 and ST78 associated with healthcare outbreaks and MDR phenotypes.^19,20^ However, the epidemiology and transmission dynamics of ECC remain poorly characterized in many African healthcare settings, including Tanzania.

This study investigates the colonization, AMR and molecular epidemiology of ECC among mothers, neonates, HCWs and hospital environments in two hospitals in the Tanga Region of Tanzania to better understand distribution patterns, potentially facilitating transmission, and inform IPC strategies.

## Methods

### Study design and study area

This cross-sectional hospital-based study was conducted in the obstetric and neonatal wards of Tanga Regional Referral Hospital (TRRH) and Korogwe Township Council Hospital (KTCH), Tanzania. TRRH is a tertiary healthcare facility with a capacity of 600 beds. The facility provides healthcare services to a population of approximately two million people. KTCH is a secondary healthcare facility situated in Korogwe District of the Tanga Region, approximately 100 km from Tanga city. The facility has a capacity of 142 beds and serves a population of approximately 300 000 people.

### Ethics

Ethical approval for this study was obtained from the National Institute for Medical Research (NIMR), Tanzania (Reference No: NIMR/HQ/R.8a/Vol.IX/3966). The study was conducted in accordance with the ethical principles outlined in the Declaration of Helsinki. Permission to conduct the study and collect samples within hospital facilities was obtained from the respective hospital administrations.

### Sample collection, ECC isolation, identification and antimicrobial susceptibility testing

Pregnant women admitted for delivery or presenting in labour were informed about the study objectives by trained study nurses, and demographic and clinical information was collected from consenting women. Neonates included infants from birth to 28 days of life with gestational age >34 weeks. Neonates with congenital malformations, stillbirth, or preterm were excluded.

Peri-anal samples were collected from mothers at admission (pre-delivery) using sterile swabs moistened with Liquid Amies medium (eSwabs 490C, Copan Diagnostics, USA). Rectal samples were obtained from neonates shortly after birth and again at discharge after ≥48 h of hospitalization. Hospital environmental samples were collected weekly using Amies swabs (Copan Diagnostics Inc.) from frequently touched surfaces, medical devices and HCWs’ hands (entire palm, including fingertips). Sampled surfaces included medication trolleys, bedside rails, keyboards and computer mice, baby warmers, cots, weighing scales, stethoscopes, thermometers, incubators and oximeters. All swabs were transported to the NIMR, Tanga Research Centre and Korogwe Research Station microbiology laboratories. Samples were enriched in 1 mL of Brain Heart Infusion Broth (Oxoid, Basingstoke, UK) and incubated for 18–24 h at 35°C–37°C and then cultured on CHROMagar^TM^ ESBL (Paris, France) and then further incubated for 18–24 h at 35°C–37°C. Suspected ESBL-producing Enterobacterales were identified using API 20E (bioMérieux, France) and confirmed using the VITEK 2 automate (VITEK GN-ID, bioMérieux). Antimicrobial susceptibility testing (AST) and ESBL confirmation were performed using the VITEK 2 Compact system (AST-N214 cards) and disc diffusion following the European Committee on Antimicrobial Susceptibility Testing (EUCAST) guidelines (Version 12, 2023). MDR was defined as resistance to greater than or equal to three antibiotic classes.^21^ EUCAST clinical breakpoints were used for the interpretation of AST results. Quality control was performed using the ECC strain ATCC 13047.

Antibiotics were classified according to the World Health Organization (WHO) Access, Watch, Reserve (AWaRe) framework using the 2023 classification.^22^

### Genome sequencing and bioinformatic analysis

#### Sequencing of bacterial isolates

DNA was extracted from pure colonies using the Master Pure Complete DNA and RNA Purification Kit (Lucigen, USA) as per the manufacturer’s instructions. DNA concentration was determined using a Qubit^TM^ 4 Fluorometer (Thermo Fisher Scientific, Singapore). Whole-genome sequencing (WGS) was performed at Beijing Genomics Institute (Hong Kong, China) using the DNBSEQ-G400 platform.

#### Assembly and sequence typing

High-quality reads were assembled in Ridom SeqSphere+ v10^23^ using the Strategic k-mer Extension for Scrupulous Assembler v2.3.0.^24^ Assembly quality was assessed using MASH (MinHash-based genome distance estimation tool) and Benchmarking Universal Single-Copy Orthologs.^25^ STs were assigned with Ridom SeqSphere+, and novel STs were deposited in PubMLST.^26^

#### Phylogenetic analysis

Phylogenetic trees were generated for *E. hormaechei* genomes using PhyloPhlAn v3.1.1^27^ with UniRef90 CORE proteins as reference (generated 4 December 2024). Trees were annotated with AMR, virulence and plasmid data and visualized in R version 4.4.2. Phylogenetic groupings are described descriptively based on ST and tree topology, without applying a predefined single-nucleotide polymorphism (SNP) threshold.

#### Temporal cluster analysis

Temporal and spatial patterns of ECC STs were assessed to identify potential transmission events. A temporal cluster was defined as greater than or equal to two isolates of the same ST recovered from the same ward within 30 days, consistent with reported survival times of Enterobacterales on hospital surfaces.^28^ Clusters were interpreted as potential persistence or transmission events; directionality could not be determined due to a lack of spatial or clinical linkage data.

#### Definitions

Colonization was defined as the presence of bacteria on body surfaces without tissue invasion or inflammatory response, whereas infection was defined as bacteria invasion into viable tissue with an inflammatory host response and clinical symptoms.

#### Data analysis

Data were collected and managed using REDCap (Vanderbilt University, USA) and Excel and analysed using Stata 18.0 (StataCorp, USA) and R 4.2.1 (R Core Team). Categorical variables were summarized as frequencies and percentages, and continuous variables as median and interquartile range (IQR). Differences between ESBL-ECC positive and negative samples were assessed using χ^2^ tests for categorical variables and Kruskal–Wallis tests for continuous variables. Logistic regression was used to identify factors associated with MDR. A *P*-value of <0.05 was considered statistically significant.

### Data availability

Raw sequencing data have been deposited in the NCBI Sequence Read Archive (SRA) under BioProject PRJNA1328667 (SRA accessions SRR35403460–SRR35403583). Genome assemblies are available in GenBank (accessions JBRQVH000000000–JBRQZZ000000000).

## Results

This study was conducted between May 2022 and March 2023. A total of 3584 samples were taken: (25%, *n*/*N* = 899/3584) from pregnant women, (23%, *n*/*N* = 826/3584) from neonates at delivery, (5%, *n*/*N* = 176/3584) from neonates at discharge, (8%, *n*/*N*, 286/3584) from the hands of HCWs and (39%, *n*/*N* = 1397/3584) from hospital surfaces including HCWs’ personal devices. Background characteristics of the samples are shown in Table 1. Significantly greater proportions of ESBL-ECC positive samples were observed from neonatal wards and from environmental samples, compared with those from labour wards and mothers and baby pairs, respectively.

**Table 1.** Description of sample sources by presence of ESBL-producing *E. cloacae* complex

	ESBL-ECC positive	Total	*P*-value
All samples			
*N*	132 (3.7%)	3584 (100.0%)	
Hospital			
TRRH	84 (4.0%)	2105 (58.7%)	0.244
KTCH	48 (3.2%)	1479 (41.3%)	
Ward			
Labour	83 (3.0%)	2740 (76.5%)	<0.001
Neonatal	49 (5.8%)	844 (23.5%)	
Hospital/ward			
TRRH/labour	50 (3.3%)	1532 (42.7%)	0.002
TRRH/neonatal	34 (5.9%)	573 (16.0%)	
KTCH/labour	33 (2.7%)	1208 (33.7%)	
KTCH/neonatal	15 (5.5%)	271 (7.6%)	
Source of sample			
Mothers and neonates	45 (2.4%)	1901 (53.0%)	<0.001
Environment	87 (5.2%)	1683 (47.0%)	
Mother samples			
*N*	11 (1.2%)	899 (100.0%)	
Age (years) (IQR)	27 (22–33)	27 (22–32)	0.793
No. of pregnancies (including current) (IQR)	3 (1–4)	2 (1–3)	0.464
Antibiotic treatment in current pregnancy			
No	3 (0.7%)	423 (47.1%)	0.130
Yes	7 (1.5%)	458 (50.9%)	
Unknown	1 (5.6%)	18 (2.0%)	
Neonate (delivery) samples			
*N*	16 (1.9%)	826 (100.0%)	
Birth weight (kg) (IQR)	3.0 (2.8–3.5)	3.0 (2.7–3.4)	0.781
Sex of neonate female	10 (2.6%)	380 (46.0%)	0.181
Caesarean section performed	7 (2.1%)	336 (40.7%)	0.801
Neonate (discharge) samples			
*N*	18 (10.2%)	176 (100.0%)	
Birth weight (kg) (IQR)	3.1 (2.7–3.6)	3.0 (2.7–3.4)	0.581
Sex of neonate Female	7 (9.0%)	78 (44.3%)	0.625
Hospital stay >48 h	18 (10.2%)	176 (100.0%)	
Caesarean section performed	17 (12.5%)	136 (77.3%)	0.067
Environmental samples			
*N*	87 (5.2%)	1683 (100.0%)	
Source of environmental samples			
HCW hands	3 (1.0%)	286 (17.0%)	<0.001
HCW devices	1 (0.4%)	271 (16.1%)	
Bedside rail	26 (9.4%)	277 (16.5%)	
Trolley	14 (8.6%)	162 (9.6%)	
Baby warmer/incubator/cot	15 (7.5%)	201 (11.9%)	
Computer keyboard/mouse	7 (4.1%)	169 (10.0%)	
Weighing scale	6 (6.1%)	98 (5.8%)	
Medical device	10 (6.0%)	166 (9.9%)	
Obstetrics theatre: any surface	5 (9.4%)	53 (3.1%)	

Categorical variable cells are *n* (%), *P*-values from χ^2^ tests. Continuous variable cells are median (IQR), *P*-values from Kruskal–Wallis rank tests.

### ECC isolated from mothers, neonates and the hospital environments

ESBL-producing ECC strains were isolated from 132 of 3584 samples (3.7%). Among human samples, 1.2% of mothers (*n*/*N* = 11/899), 1.9% of neonates at delivery (*n*/*N* = 16/826) and 10.2% of neonates at discharge after >48 h of hospitalization (*n*/*N* = 18/176) were positive. Among environmental samples, including HCWs’ hands, 5.2% were positive (*n*/*N* = 87/1683). The lowest detection rates were observed on HCWs’ hands (1.0%, *n*/*N* = 3/286) and personal devices (0.4%, *n*/*N* = 1/271). In contrast, hospital surfaces showed higher positivity, ranging from 4.1% on computer keyboards and mice (*n*/*N* = 7/169) to 9.4% on bedside rails (*n*/*N* = 26/277) and obstetric theatre surfaces (*n*/*N* = 5/53).

**Figure 1.**
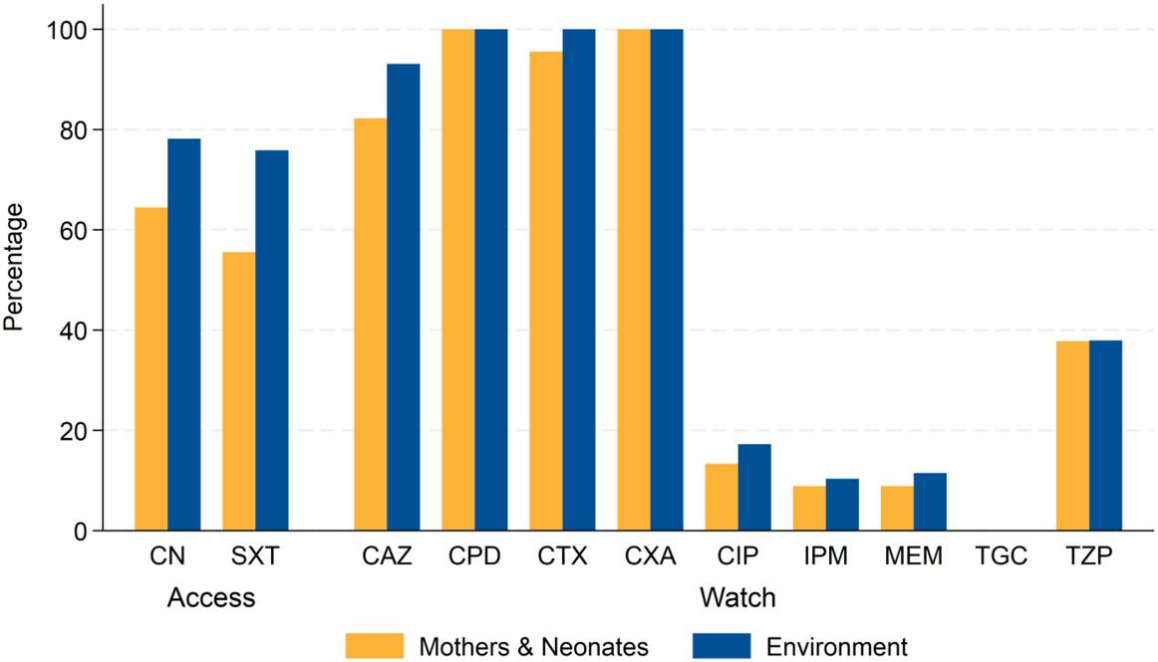
AMR for ESBL-producing ECC isolated from mothers and neonates, and the hospital environment, sorted by Access and Watch agents. CN, gentamicin; SXT, trimethoprim–sulfamethoxazole; CAZ, ceftazidime; CPD, cefpodoxime; CTX, cefotaxime; CXA, cefuroxime-axetil; CIP, ciprofloxacin; IPM, imipenem; MEM, meropenem; TGC; tigecycline; TZP, piperacillin–tazobactam. Access & Watch, AWaRe categories of antibiotics as per the WHO 2023 classification^22^; Access and Watch shown; reserve not tested.

AMR profiles of ESBL-producing ECC isolates from human and environmental sources were compared (Figure 1). Resistance in environmental isolates was generally higher or comparable to that in human isolates, reaching statistical significance only for trimethoprim–sulfamethoxazole (χ^2^  *P* = 0.017). Resistance to second- and third-generation cephalosporins (ceftazidime, cefpodoxime, cefotaxime and cefuroxime-axetil) exceeded 89.4% (*n*/*N* = 118/132–132/132). No resistance was detected to tigecycline, while lower resistance levels were observed for carbapenems (10.6%, *n*/*N* = 14/132) and ciprofloxacin (15.9%, *n*/*N* = 21/132).

### MDR levels across sample sources and wards

**Figure 2.**
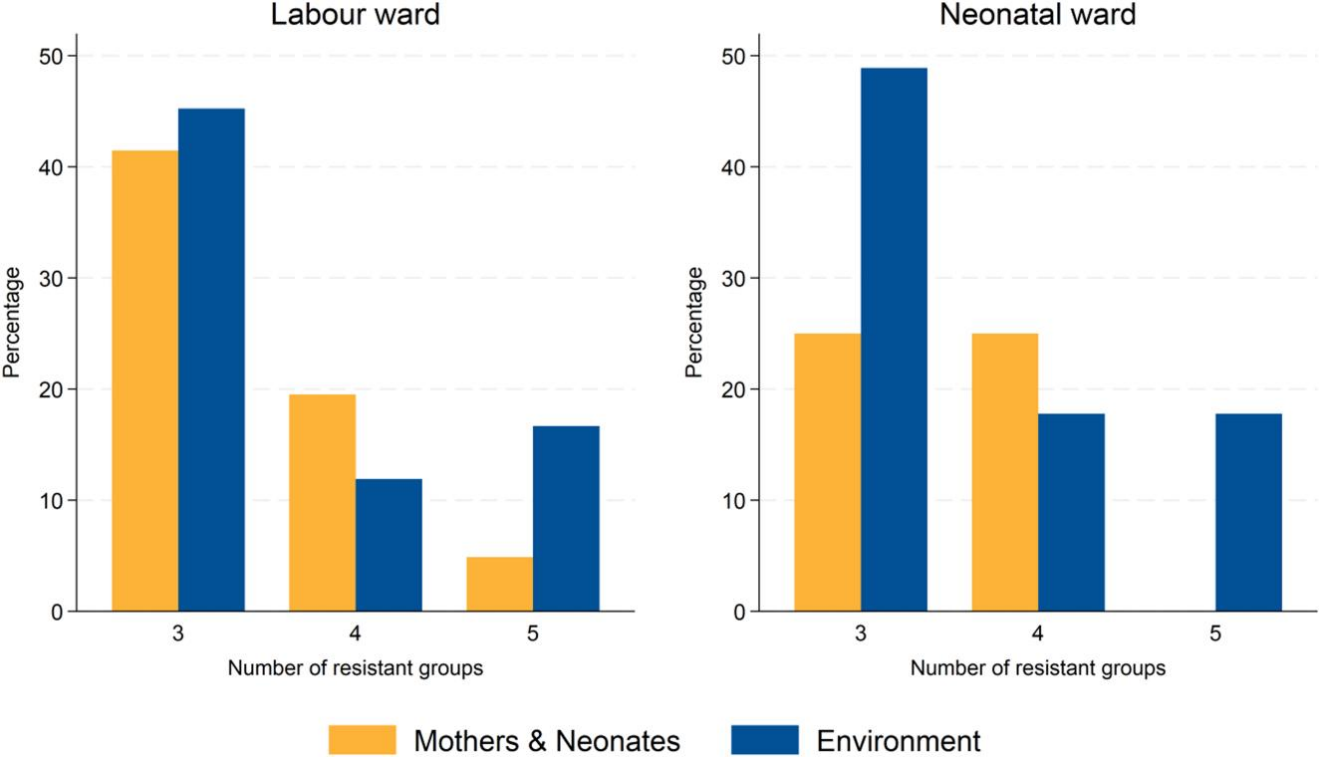
MDR levels (Levels 3–5) across sample source and ward; percentages based on the total number of ESBL-ECC samples in each source/ward category in both hospitals.

Overall, 74.2% (*n*/*N* = 98/132) of ESBL-producing ECC isolates were MDR (Figure 2). MDR was more frequent in neonatal wards (81.6%, *n*/*N* = 40/49) than in labour wards (69.9%, *n*/*N* = 58/83), a pattern observed in both hospitals. Similarly, MDR prevalence was higher in environmental samples (79.3%, *n*/*N* = 69/87) than in samples from mothers and neonates (64.4%, *n*/*N* = 29/45).

Adjusted logistic regression analysis showed increased odds of MDR in isolates from neonatal wards [odds ratio (OR) = 1.50, 95% confidence interval (CI): 0.58–3.90] and from the hospital environment (OR = 1.79, 95% CI: 0.74–4.34). However, these associations were not statistically significant (Table S2a, available as Supplementary data at *JAC-AMR* Online).

### Species distribution and genome assembly characteristics

Of 132 sequenced genomes, 12 were excluded due to low coverage, contamination, or incomplete assemblies, leaving 120 high-quality ECC genomes. These were dominated by *E. hormaechei* (*n* = 112), with *E. cloacae* (*n* = 5) and single isolates of *Enterobacter hoffmannii*, *Enterobacter quasihormaechei* and *Enterobacter vonholyi*.

Across the five species, sequencing depth was high (mean coverage ×180 to ×210). Assemblies showed moderate fragmentation (28–189 contigs; N50 78–537 kb), with conserved guanine-cytosine (GC) content (54.7%–55.9%). Genome sizes ranged from 4.5 to 5.1 Mb, with *E. quasihormaechei* having the smallest and *E. cloacae* the largest genomes (Table S1).

### Distribution of antimicrobial genes

A total of 22 beta-lactamase genes were identified. The *bla_CTX-M_* group was most common (89.2%, *n*/*N* = 107/120), dominated by *bla*_CTX-M-15_ (87.5%, *n*/*N* = 105/120). Other frequently detected genes included blaTEM-1 (60.8%, *n*/*N* = 73/120) and *bla*_OXA-1_ (49.2%, *n*/*N* = 59/120). Chromosomal *bla*_ACT_ genes were present in 95.8% of isolates. Carbapenem resistance genes were detected in 10.8%, including *bla*_NDM-1_, *bla*_NDM-5_ and *bla*_OXA-181_ (Table S2a).

### Other resistance genes

Resistance determinants for multiple antimicrobial classes were identified. Common genes included *fosA* (91.7%, *n*/*N* = 110/120), aminoglycoside resistance genes *aph(6)-Id* and *aph(3'‘)-Ib* (68.3%, *n*/*N* = 82/120), sulphonamide genes *sul2* (69.2%, *n*/*N* = 83/120) and fluoroquinolone resistance genes *oqxAB* (90%, *n*/*N* = 108/120) and *qnrB1* (74.2%, *n*/*N* = 90/120). Additional genes conferring resistance to tetracycline, trimethoprim, colistin (*mcr*-10.1), macrolides, rifamycin and bleomycin were also detected. Furthermore, the *qacE* and *qacE delta 1* resistance genes for quaternary ammonium compound-related disinfectants were also detected (12.1%, *n*/*N* = 15/124) (Table S2b).

### Localization of resistance genes

A total of 1492 AMR genes were identified across the genomes. Several genes showed distinct genomic locations: *bla*_TEM-1_ and sulphonamide genes were predominantly plasmid-borne, whereas *bla*_ACT_, *bla*_CMH_ and *fosA* were chromosomal. *bla*_CTX-M-15_ was mainly plasmid-associated, while *bla*_OXA-1_ occurred more frequently on chromosomes (Table S3).

### Genotype–phenotype correlation of AMR

All 120 isolates carried β-lactam resistance genes and were also phenotypically resistant (100% concordance). Carbapenem resistance genes were identified in 12 isolates, all phenotypically resistant, although one additional isolate showed carbapenem resistance without a detectable gene. Fluoroquinolone resistance genes were common (118/120), but phenotypic resistance was observed in only 21 isolates (Table 2).

**Table 2.** Comparison of genotypic resistance genes and phenotypic resistance

Antibiotic class tested	Resistance genes detected (genotypic)	Isolates carrying resistance genes	Phenotypically resistant among gene carriers	Phenotypically resistant isolates	Genotypically confirmed resistant isolates
Non-carbapenem β-lactams (penicillins and cephalosporins)	*bla*_CTX-M-15_ *, bla*_CTX-M-14_ *, bla*_TEM-1_ *, bla*_OXA-1_ *, bla*_SHV-12_ *, bla*_ACT_ *, bla*_CMH_	120	120	120	120
Carbapenems	*bla*_NDM-1_ *, bla*_NDM-5_ *, bla*_OXA-181_	12	12	13	12
Aminoglycosides	*aac(3)-IId, aac(3)-IIe, aac(6‘)-Ib-cr5 aph(3“)-Ib, aph(6)Id, aadA1, aadA2, aadA16*	107	88	75	75
Fluoroquinolones	*qnrS1, qnrB1, qnrB4, oqxA/oqxB*	118	21	21	21
Sulphonamides + diaminopyrimines	sul1/sul2*, dfrA*	106	106	120	106

Phenotypically resistant among gene carriers = number of gene-carrying isolates that also showed phenotypic resistance. Genotypically confirmed resistant isolates = number of phenotypically resistant isolates that also had corresponding resistance genes. Comparison done with 120 sequenced isolates.

### ST distribution

Among the 120 ECC isolates, 41 STs were identified, including twelve novel STs (10%, *n*/*N* = 12/120). ST346 was the most common (25%, *n*/*N* = 30/120), followed by ST2713 and ST109 (8.3%, *n*/*N* = 10/120 each), ST66 (7.5%, *n*/*N* = 9/120) and ST171 (4.1%, *n*/*N* = 5/120). ST346 predominated in both environmental (28.6%, *n*/*N* = 22/77) and human isolates (18.6%, *n*/*N* = 8/43). Other STs were more source-specific, with ST66 and ST171 mainly environmental, while ST2713 was more frequent in human isolates (11.6%, *n*/*N* = 5/43) (Table S4).

### Phylogenetic analysis and associated genetic profiles of *E*. *hormaechei* isolates

**Figure 3.**
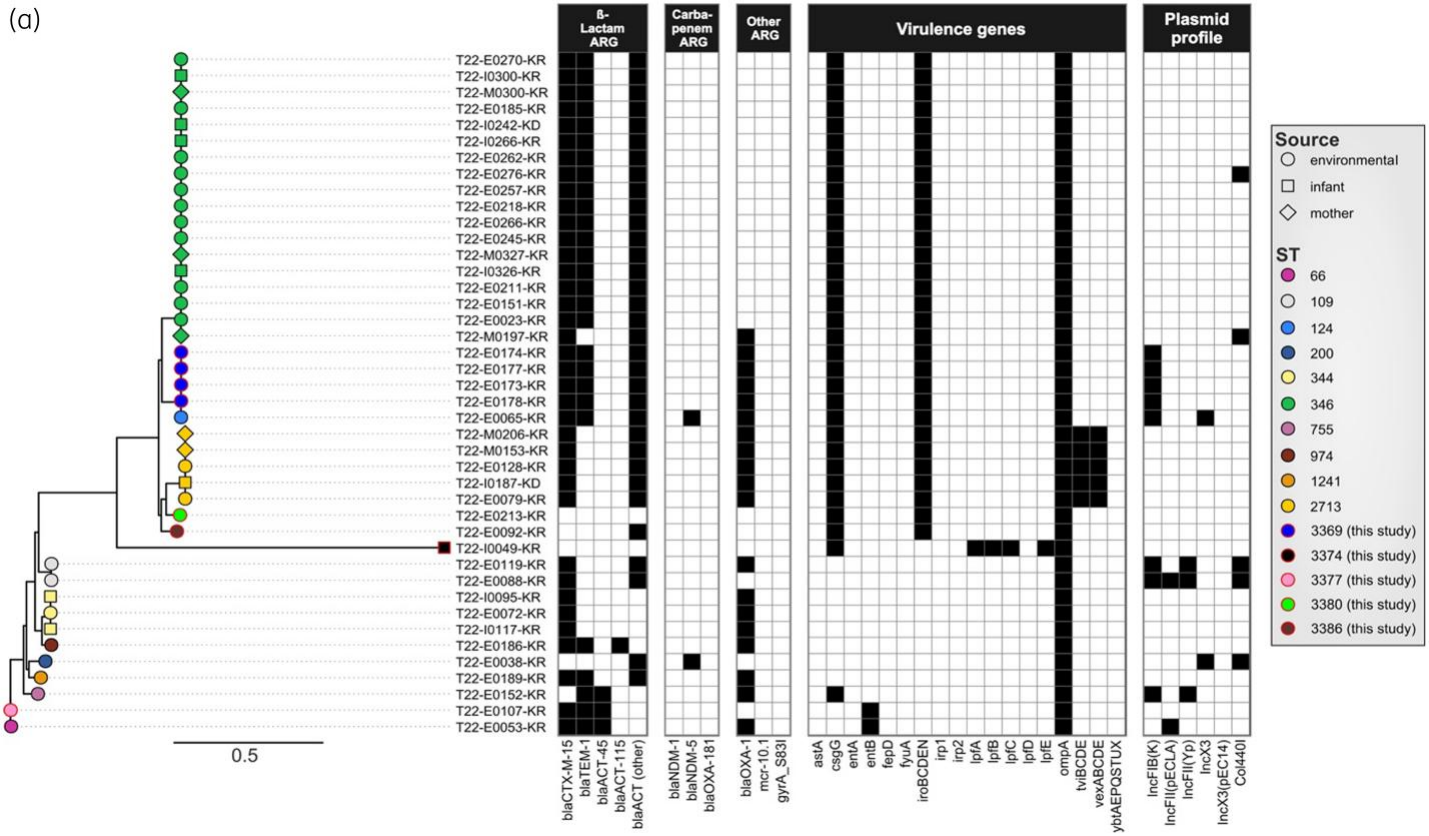

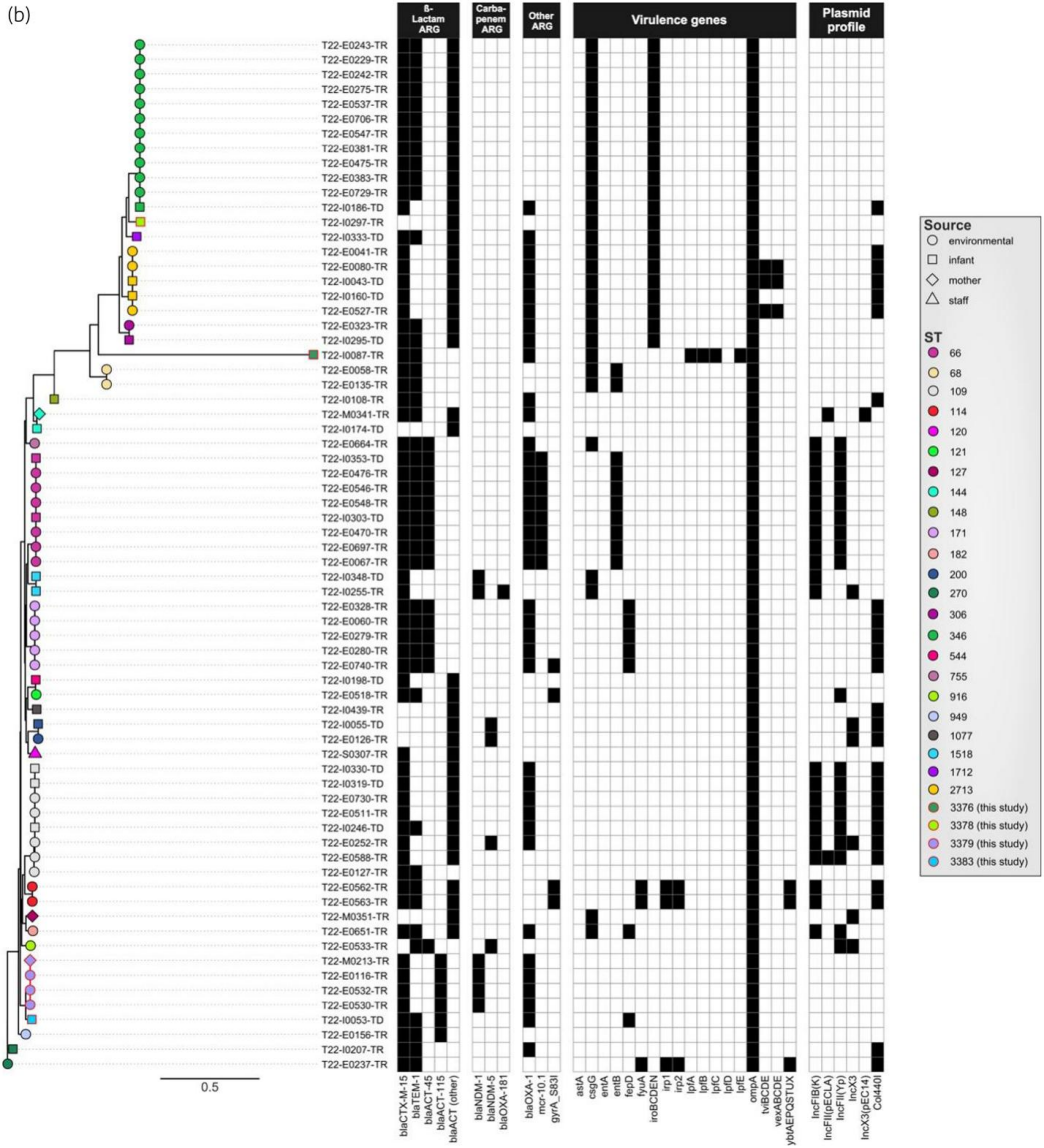
Phylogenetic relationship and genetic profiles of *E. hormaechei* isolates collected from mothers, neonates, the hospital environment and staff at KTCH (a) and TRRH (b). The dendrogram illustrates the phylogenetic relatedness of isolates, while the presence (black) and absence (white) of key β-lactamase genes, carbapenemase genes, virulence genes and plasmid sequences are displayed in corresponding columns. Isolate sources and STs are indicated by different shapes and colours, respectively.

Phylogenetic analysis showed that *E. hormaechei* isolates clustered according to ST, forming distinct phylogenetic groupings with isolates from different sources interspersed within clusters (Figure 3a and b).

At KTCH (Figure 3a), ST346 was the dominant lineage (42.9%; *n*/*N* = 18/42) and included isolates from all sources, including a closely related mother–infant pair. All ST346 isolates carried the ESBL determinant (*bla*_CTX-M-15_) and consistently harboured virulence genes associated with biofilm formation and iron acquisition (*csgG*, *iroABCD*) as well as adhesion and colonization (*ompA*). Notably, carbapenem resistance genes (*bla*_NDM-5_) were identified in isolates belonging to ST124 and ST200, both associated with IncX3 plasmids.

At TRRH (Figure 3b), a more diverse phylogenetic structure was observed. ST346 was also the dominant lineage (17.1%; *n*/*N* = 12/70) and showed a resistance and virulence profile similar to that observed at KTCH, including carriage of the ESBL and virulence-associated genes. Several additional ST-associated clusters displayed distinct resistance profiles. ST66 accounted for 11.4% (*n*/*N* = 8/70) of isolates and formed a well-defined cluster characterized by carriage of colistin resistance genes (*mcr-*10.1), ESBL determinants (*bla*_CTX-M-15_), and virulence-associated genes involved in iron acquisition and adhesion (*entB*, *ompA*). Notably, a separate ST3379 cluster carried carbapenem resistance genes (*bla*_NDM-1_) together with resistance determinants spanning eight additional antimicrobial classes (Table S5). ST1518 isolates formed a closely related cluster, with both isolates carrying carbapenem resistance genes (*bla*_NDM-1_) and one additionally harbouring *bla*_OXA-181_. Carbapenem resistance genes were also identified in additional STs, including ST200, ST916 and ST344.

Across both hospitals, ST2713 formed a distinct phylogenetic cluster, comprising 11.9% (*n*/*N* = 5/42) of isolates at KTCH and 7.1% (*n*/*N* = 5/70) at TRRH. All ST2713 isolates from TRRH carried virulence operons associated with capsule biosynthesis and export (*tviBCDE* and *vexABCDE*), whereas three of five corresponding isolates from KTCH harboured these operons.

### Temporal clustering of ECC isolates

**Figure 4.**
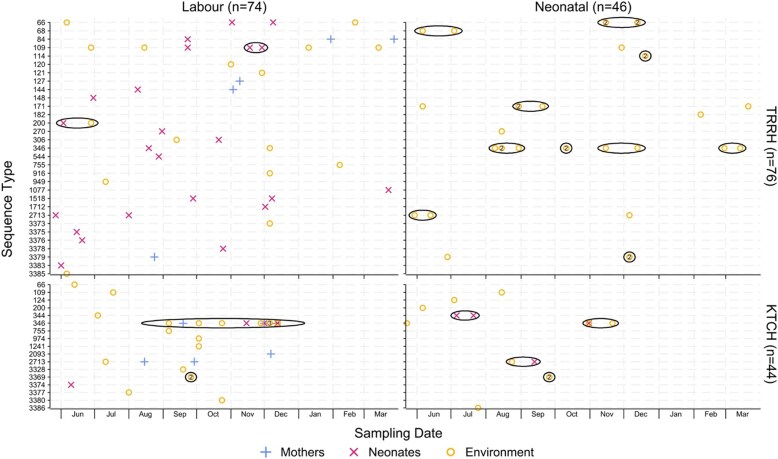
Temporal distribution of STs in labour and neonatal wards of TRRH and KTCH. Phylogenetically closely related ST clusters occurring within a 30 day time frame (highlighted by ovals) were considered potential transmission events.

Temporal patterns of ECC recovery were assessed to identify potential transmission or persistence within wards at TRRH and KTCH (Figure 4). In the labour ward of TRRH, temporal clusters were observed for ST109 (two neonatal isolates) and ST200, which was detected first in a neonate and within 30 days in the environment. In the neonatal ward, clusters involved only environmental isolates (ST66, ST68 and ST114). ST346 also formed four distinct temporal clusters in this ward.

At KTCH, a large ST346 cluster in the labour ward involved 14 isolates, including a mother-neonate pair detected on the same day. Another cluster (ST3369) included two environmental isolates. In the neonatal ward, clusters were observed for ST344 (two neonatal isolates) and ST346 (environmental and neonatal isolates detected on the same day). Additional clusters included ST2713, first detected in the environment and within 30 days in a neonate, and ST3369 (both environmental isolates).

### Virulence genes and plasmid incompatibility types

Several virulence-associated genes were detected among the 120 ECC isolates (Table S6). The adhesion gene ompA was present in all isolates (100%). Biofilm formation gene csgG (55.8%) and the iron acquisition operon iroBCDEN (42.5%) were the most frequent additional virulence determinants.

Seventeen distinct plasmid incompatibility (Inc) types were identified among the 120 ECC isolates (Table S7). The most frequent were IncR (37.5%), IncFIB(K) (27.5%) and Col440I (27.5%), followed by IncFIA(HI1) (25%) and IncFII(Yp) (20.8%). Other plasmid types, including IncHI2/IncHI2A (14.2%), IncX3 (6.6%) and IncFIB(pECLA) (5.8%), were detected at lower frequencies, while several additional types occurred only sporadically (<5%).

## Discussion

This study provides insights into the genetic epidemiology and potential transmission dynamics of ESBL-producing ECC in neonatal and labour wards in two Tanzanian hospitals. We observed frequent colonization of neonates and hospital surfaces, accompanied by substantial genomic diversity, including 12 previously undescribed STs. Clonal clustering across wards, sources and time points suggests persistence and likely intra-hospital transmission of MDR ECC within these healthcare settings.

This study showed that neonatal colonization with ESBL-ECC was low at delivery (1.9%) but increased to 10.2% after 48 h of hospitalization, suggesting hospital-associated acquisition. Similar patterns have been observed in tertiary hospitals in Tanzania and South Africa,^16,29,30^ highlighting the likely role of the hospital environment in neonatal colonization. While post-partum colonization with bacteria is *a priori* a physiological process, it bears a risk for neonates of developing subsequent infections in case of colonization with bacteria showing increased virulence or in case of reduced immunocompetence, including sepsis, which is associated with significant morbidity and mortality.^28^ If bacteria causing such infections are associated with increased AMR, the therapeutic management of respective infections will become more challenging. Therefore, the detection of increased resistance rates calls for a strengthening of IPC measures in the studied facilities, including hand hygiene, environmental decontamination and targeted surveillance strategies in high-risk neonatal units.^31^

Environmental samples showed a higher occurrence of ESBL-ECC (5.2%) than the human samples (2.4%). This is consistent with previous reports from SSA^32–34^ and supports the role of environmental reservoirs in pathogen persistence. Although HCW hand contamination was low (1.0%), previous studies highlight their role in pathogen transmission, underscoring the need for continued reinforcement of hand hygiene and infection prevention practices.^35–37^

Interestingly, most neonates colonized at birth had mothers without detectable colonization, suggesting colonization below detection limits or alternative sources of early microbial acquisition. Previous studies link neonatal colonization to delivery mode, gestational age, feeding practices and environmental exposure.^38–40^ These findings highlight the importance of rigorous environmental hygiene immediately after delivery.

AMR among ECC isolates was substantial, with most isolates resistant to third-generation cephalosporins due to ESBL production. Notably, 10.8% of isolates carried carbapenem resistance genes, consistent with previous observations in Tanzanian neonatal units.^41^ Environmental isolates showed higher MDR rates than human isolates, supporting the role of hospital surfaces as reservoirs for resistant strains, in line with reports from Tanzania, Kenya and Pakistan.^32,33,42^ MDR was more frequent in neonatal wards than labour wards, possibly reflecting more intensive antibiotic use in high-risk neonates.^16,43^

Genomic analysis revealed widespread resistance determinants across multiple antibiotic classes. The ESBL gene *bla*_CTX-M-15_ and chromosomal *ampC*-type *bla*_ACT_ genes were highly prevalent, consistent with global ECC resistance patterns.^44^ Notably, carbapenemase genes were detected in ECC isolates from both neonatal and environmental samples. While NewDelhi metallo-beta-lactamase (NDM)-type carbapenemases have previously been reported in Enterobacterales from Tanzanian neonatal units,^30^ our findings demonstrate their occurrence in ECC and in hospital environmental isolates, suggesting the hospital environment as a possible reservoir for dissemination. Moreover, the detection of recently identified colistin resistance gene *mcr-*10.1 in a subset of isolates represents one of the first reports from Tanzania,^45^ reflecting the potential emergence of resistance to last-resort antibiotics. In addition, resistance genes for aminoglycosides, sulphonamides and fluoroquinolones were widespread, highlighting the MDR nature of the isolates and the potential for horizontal gene transfer and dissemination within hospital environments.

The present study has also demonstrated the presence of *qacE and qacE delta 1* genes. These genes are known to reduce susceptibility to quaternary ammonium compounds (QACs), which are commonly used disinfectants in healthcare settings, thereby potentially compromising the IPC measures.^46^

Phenotype–genotype comparison showed full concordance for β-lactams and carbapenems, whereas aminoglycosides and fluoroquinolones displayed discordance, with resistance genes present but lacking phenotypic resistance. This likely reflects low-level plasmid-mediated mechanisms (e.g. *qnr*, *oqx*, aminoglycoside-modifying enzymes) that do not exceed clinical breakpoints without additional mutations or expression changes.^47–49^ Such a discrepancy can lead to treatment failure as the low-level resistance mechanisms may evolve to induce high-level resistance during the course of treatment; furthermore, the genes may be transmissible, and strains are also able to spread, increasing the risk of AMR.^50^

Molecular typing revealed substantial ST diversity, with ST346 emerging as the most dominant lineage (25%), across both hospitals and sample sources. Its repeated detection on frequently touched surfaces and in patients over time may indicate persistence and potential environmental transmission within hospital settings. Globally, ST346 has been rarely reported,^51^ indicating the presence of locally adapted hospital lineages. Notably, all ST346 isolates were MDR and carried ESBL genes, highlighting significant infection control challenges.

Another noteworthy lineage, ST2713, was uniquely associated with the virulence operons, *tviBCDE* and *vexABCDE,*^52^ implicated in immune evasion and increased virulence.^53^ Temporal clustering of ST2713 isolates was also observed, suggesting enhanced pathogenic potential and persistence within the hospital environment.

Interestingly, detection of chromosomal *mcr-*10.1 exclusively occurred within ST66 isolates, suggesting a lineage-specific association. Although *mcr-*10.1 has been sporadically reported in ECC globally,^54^ to the best of our knowledge, this study provides the first evidence of its lineage-specific association with ST66 in a Tanzanian hospital setting. These isolates also occurred within temporally clustered groups, highlighting the emergence of colistin resistance in circulating hospital lineages.

We detected carbapenem resistance genes in several ST-defined lineages, including STs previously associated with carbapenemase carriage (ST109, ST121, ST1518, ST124 and ST200), indicating the circulation of lineages known to harbour carbapenem resistance.^7,19^ Notably, ST1518 lineage carried both *bla*_NDM-1_ and *bla*_OXA-181_, representing a rare dual-carbapenemase genotype in ECC. Intriguingly, all positive *bla*_NDM-5_ isolates carried IncX3 plasmids, known globally for efficient horizontal transfer of *bla*_NDM_ and *bla*_OXA_ across Enterobacterales.^55^ A newly identified ST3379 lineage at TRRH carried *bla*_NDM-1_ together with resistance genes to eight additional antibiotic classes, indicating a highly resistant profile and raising concerns about the emergence of such strains in neonatal wards.

This study has several limitations. The WGS analysis has been performed to the level of ST, which is not sufficient to prove cross-contamination from the hospital environment. The use of higher-resolution analyses, such as the SNP, could have provided stronger evidence on potential transmission pathways. However, without clear-cut epidemiological evidence, transmission events remain speculative. In addition, the study was conducted in only two hospitals in one region of Tanzania, which may limit generalizability. Finally, colonized neonates were not followed to assess subsequent infections such as late-onset sepsis. Future studies should evaluate the clinical impact of intestinal colonization with resistant ECC and determine whether these strains contribute to infection or persist primarily as commensal colonizers.

### Conclusions

This study provides molecular and epidemiological characterization of MDR ECC colonization across mothers, neonates, HCWs and hospital environments in two hospitals in Tanga, Tanzania. We identified 12 novel ECC STs, indicating substantial genomic diversification. The dominance and persistence of ST346 among patients and frequently touched environmental surfaces highlight the role of the hospital environment as a possible reservoir facilitating potential transmission events. Detection of ESBL and carbapenem resistance genes, including *bla*_CTX-M-15_ and *bla*_NDM_, as well as the colistin resistance gene *mcr*-10.1 within temporally and phylogenetically related isolates, underscores the risk of intra-hospital spread of MDR and extensively drug-resistant strains. These findings emphasize the need for strengthened IPC measures, targeted environmental decontamination and sustained genomic surveillance to limit the dissemination of MDR ECC in neonatal care settings.

## Supplementary Material

dlag161_Supplementary_Data

## Supplementary data

Tables S1–S7 are available as Supplementary data at *JAC-AMR* Online.

## References

[1] De Maayer P, Green T, Jordan S et al Pan-genome analysis of the *Enterobacter hormaechei* complex highlights its genomic flexibility and pertinence as a multidrug resistant pathogen. BMC Genomics 2025; 26: 408. 10.1186/s12864-025-11590-1 40287657 PMC12034153

[2] Mauritz MD, Claus B, Forster J et al The EC-COMPASS: long-term, multi-centre surveillance of *Enterobacter cloacae* complex—a clinical perspective. J Hosp Infect 2024; 148: 11–9. 10.1016/j.jhin.2024.03.010 38554809

[3] Han M, Hua M, Xie H et al Clinical characteristics and risk factors for multidrug-resistant *Enterobacter cloacae* complex bacteremia in a Chinese Tertiary Hospital: a decade review (2013–2022). Infect Drug Resist 2025; 18: 427–40. 10.2147/IDR.S502509 39867289 PMC11766150

[4] Cia C-T, Su S-L, Tsai P-F et al Infections caused by clonal spread of metallo-beta-lactamase-producing *Enterobacter cloacae* complex isolates at a southern Taiwan hospital. Microbiol Spectr 2025; 13: e0023425. 10.1128/spectrum.00234-25 40492759 PMC12210983

[5] Mansouri S, Savari M, Malakian A et al High prevalence of multidrug-resistant Enterobacterales carrying extended-spectrum beta-lactamase and AmpC genes isolated from neonatal sepsis in Ahvaz, Iran. BMC Microbiol 2024; 24: 136. 10.1186/s12866-024-03285-6 38658819 PMC11040821

[6] Boattini M, Bianco G, Llorente LI et al Enterobacterales carrying chromosomal AmpC β-lactamases in Europe (EuESCPM): epidemiology and antimicrobial resistance burden from a cohort of 27 hospitals, 2020–2022. Int J Antimicrob Agents 2024; 63: 107115. 10.1016/j.ijantimicag.2024.107115 38367844

[7] Cai S, Quan J, Wang Z et al High prevalence of carbapenem-resistant *Enterobacter cloacae* complex in a tertiary hospital over a decade. Microbiol Spectr 2024; 12: e0078024. 10.1128/spectrum.00780-24 39475294 PMC11619405

[8] Lakshmanan D, Ramasamy D, Subramanyam V et al Mobile colistin resistance (mcr) genes and recent developments in colistin resistance detection. Lett Appl Microbiol 2023; 76: ovad102. 10.1093/lambio/ovad102 37673673

[9] Afsharian M, Asadi S, Danesh C et al The abundance of plasmid-mediated quinolone resistance genes in *Enterobacter cloacae* strains isolated from clinical specimens in Kermanshah. Can J Infect Dis Med Microbiol 2024; 2024: 8849097. 10.1155/2024/8849097 38623587 PMC11018368

[10] Kidenya BR, Mboowa G, Sserwadda I et al Whole genome-based characterization of extended-spectrum β-lactamase-producing *Enterobacter cloacae* from orthopedic patients and environment of a tertiary referral hospital in Tanzania. New Microbes New Infect 2024; 62: 101486. 10.1016/j.nmni.2024.101486 39386352 PMC11462357

[11] Wang X, Tian Y, Zhang Q et al Bloodstream infection with NDM-1/5 *Enterobacter cloacae* complex in China: diverse STs, multi-virulence systems and carbapenem resistance. Front Cell Infect Microbiol 2026; 15: 1738317. 10.3389/fcimb.2025.1738317 41613598 PMC12847444

[12] Perez-Palacios P, Girlich D, Soraa N et al Multidrug-resistant Enterobacterales responsible for septicaemia in a neonatal intensive care unit in Morocco. J Glob Antimicrob Resist 2023; 33: 208–17. 10.1016/j.jgar.2023.02.011 36868310

[13] Fernández-Martínez NF, Rivera-Izquierdo M, Ortiz-González-Serna R et al Healthcare-associated infections by multidrug-resistant bacteria in Andalusia, Spain, 2014 to 2021. Euro Surveill 2023; 28: 2200805. 10.2807/1560-7917.ES.2023.28.39.2200805 37768559 PMC10540512

[14] Iqbal F, Siva N, Shenoy PA et al Gut pathogen colonization: a risk factor to bloodstream infections in preterm neonates admitted in the neonatal intensive care unit—a prospective cohort study. Neonatology 2024; 122: 151–60. 10.1159/000542335 39675351 PMC11965847

[15] Osei Sekyere J, Mmatli M, Bosch A et al Molecular epidemiology of multidrug-resistant *Klebsiella pneumoniae*, *Enterobacter cloacae*, and *Escherichia coli* outbreak among neonates in Tembisa hospital, South Africa. Front Cell Infect Microbiol 2024; 14: 1328123. 10.3389/fcimb.2024.1328123 38481664 PMC10933102

[16] Mntla N, Chibabhai V, Nana T. Prevalence of multidrug-resistant organisms colonizing neonates at a tertiary hospital in Johannesburg, South Africa. J Trop Pediatr 2026; 72: fmaf051. 10.1093/tropej/fmaf051 41481359 PMC12758378

[17] Elton L, Williams A, Ali S et al Tracing the transmission of carbapenem-resistant Enterobacterales at the patient: ward environmental nexus. Ann Clin Microbiol Antimicrob 2024; 23: 108. 10.1186/s12941-024-00762-8 39707381 PMC11662836

[18] Nizam Ahmed M, Puraswani M, Singh P et al P44 multi-source environmental reservoirs drive *Enterobacter cloacae* complex transmission: genomic evidence from an LMIC hospital outbreak investigation. JAC Antimicrob Resist 2025; 7: dlaf230.051. 10.1093/jacamr/dlaf230.051

[19] Ye K, Zhang Y, Qiu X et al Surveillance and characterization of carbapenem-resistant *Enterobacter cloacae* complex from China, 2015–2018. BMC Microbiol 2025; 25: 597. 10.1186/s12866-025-04299-4 41039208 PMC12492719

[20] Ghazawi A, Anes F, Mouftah S et al Genomic study of high-risk clones of *Enterobacter hormaechei* collected from tertiary hospitals in the United Arab Emirates. Antibiotics 2024; 13: 592. 10.3390/antibiotics13070592 39061274 PMC11274081

[21] Birlutiu V, Birlutiu R-M. An overview of the epidemiology of multidrug resistance and bacterial resistance mechanisms: what solutions are available? A comprehensive review. Microorganisms 2025; 13: 2194. 10.3390/microorganisms13092194 41011524 PMC12472688

[22] World Health Organization . AWaRe Classification of Antibiotics for Evaluation and Monitoring of Use. World Health Organization; 2023.

[23] Jünemann S, Sedlazeck FJ, Prior K et al Updating benchtop sequencing performance comparison. Nat Biotechnol 2013; 31: 294–6. 10.1038/nbt.2522 23563421

[24] Souvorov A, Agarwala R, Lipman DJ. SKESA: strategic k-mer extension for scrupulous assemblies. Genome Biol 2018; 19: 153. 10.1186/s13059-018-1540-z 30286803 PMC6172800

[25] Tegenfeldt F, Kuznetsov D, Manni M et al OrthoDB and BUSCO update: annotation of orthologs with wider sampling of genomes. Nucleic Acids Res 2025; 53: D516–22. 10.1093/nar/gkae987 39535043 PMC11701741

[26] Jolley KA, Bray JE, Maiden MCJ. Open-access bacterial population genomics: BIGSdb software, the PubMLST.org website and their applications. Wellcome Open Res 2018; 3: 124. 10.12688/wellcomeopenres.14826.1 30345391 PMC6192448

[27] Asnicar F, Thomas AM, Beghini F et al Precise phylogenetic analysis of microbial isolates and genomes from metagenomes using PhyloPhlAn 3.0. Nat Commun 2020; 11: 2500. 10.1038/s41467-020-16366-7 32427907 PMC7237447

[28] Kramer A, Lexow F, Bludau A et al How long do bacteria, fungi, protozoa, and viruses retain their replication capacity on inanimate surfaces? A systematic review examining environmental resilience versus healthcare-associated infection risk by “fomite-borne risk assessment”. Clin Microbiol Rev 2024; 37: e0018623. 10.1128/cmr.00186-23 39388143 PMC11640306

[29] Salega HA, Kamori D, Kibwana UO et al Faecal carriage of multidrug resistant enterobacterales and associated factors among neonates admitted at Tertiary Hospital in Dar es Salaam, Tanzania. East Afr Health Res J 2025; 8: 346–53. 10.24248/eahrj.v8i3.804 PMC12542991 41132311

[30] Naburi H, Sewunet T, Tellapragada C et al Emergence of carbapenem-producing enterobacteriaceae (CPE) and other multidrug-resistant gram-negative bacteria in neonates at a tertiary-level NICU in Tanzania: a point prevalence study. JAC Antimicrob Resist 2025; 7: dlaf179. 10.1093/jacamr/dlaf179 41113065 PMC12528853

[31] Vasave U, Paroha A. Customizing infection prevention and control modules for combating healthcare-acquired infections in low-resource hospitals or resource-constrained healthcare settings : a local and global approach. Antimicrob Resist Infect Control 2026; 15: 58. 10.1186/s13756-026-01727-6 41814356 PMC13094052

[32] Gamuya E, Salum MS, Mtewele BA et al Contamination of hospital surfaces by third-generation cephalosporin-resistant gram-negative bacteria in district hospitals in Mwanza, Tanzania: urgent need for enhanced infection prevention and control. Infect Prev Pract 2025; 7: 100475. 10.1016/j.infpip.2025.100475 40922938 PMC12414270

[33] Odoyo E, Matano D, Tiria F et al Environmental contamination across multiple hospital departments with multidrug-resistant bacteria pose an elevated risk of healthcare-associated infections in Kenyan hospitals. Antimicrob Resist Infect Control 2023; 12: 22. 10.1186/s13756-023-01227-x 36978195 PMC10053033

[34] Odoi H, Dodoo CC, Gbemu MJ et al Surveillance on antimicrobial resistance: bacteriological monitoring and resistance profiles of isolates from surfaces and equipment at a Ghanaian tertiary healthcare facility. BMC Infect Dis 2025; 25: 1272. 10.1186/s12879-025-11680-1 41073979 PMC12512875

[35] Asare Yeboah EE, Agyepong N, Mbanga J et al Multidrug-resistant Gram-negative bacterial colonization in patients, carriage by healthcare workers and contamination of hospital environments in Ghana. J Infect Public Health 2023; 16: 2–8. 10.1016/j.jiph.2023.10.045 37953109

[36] Asif S, Khan MA, Basheer V et al Longitudinal evaluation of a multimodal hand hygiene intervention in improving healthcare worker compliance: a three-year quasi-experimental study at a cardiac specialty hospital in Karachi, Pakistan. Antimicrob Resist Infect Control 2026; 15: 20. 10.1186/s13756-025-01689-1 41547880 PMC12895693

[37] Amin Sumon MS, Masuda Akther F, Tamanna T et al P-1101. Can targeted interventions improve the hand hygiene compliance among healthcare workers in low resource settings? Open Forum Infect Dis 2026; 13: ofaf695.1296. 10.1093/ofid/ofaf695.1296

[38] Kennedy KM, Plagemann A, Sommer J et al Delivery mode, birth order, and sex impact neonatal microbial colonization. Gut Microbes 2025; 17: 2491667. 10.1080/19490976.2025.2491667 40251947 PMC12013413

[39] Ronde E, Alkema M, Dierikx T et al The influence of maternal gut and vaginal microbiota on gastrointestinal colonization of neonates born vaginally and per caesarean section. BMC Pregnancy Childbirth 2025; 25: 254. 10.1186/s12884-025-07358-w 40057706 PMC11889873

[40] Linehan K, Healy K, Hurley E et al Perinatal factors influencing the earliest establishment of the infant microbiome. Microbiome Res Rep 2025; 4: 24. 10.20517/mrr.2024.92 40852123 PMC12369391

[41] Silago V, Kovacs D, Msanga DR et al Bacteremia in critical care units at Bugando Medical Centre, Mwanza, Tanzania: the role of colonization and contaminated cots and mothers’ hands in cross-transmission of multidrug resistant Gram-negative bacteria. Antimicrob Resist Infect Control 2020; 9: 58. 10.1186/s13756-020-00721-w 32375857 PMC7201549

[42] Hussain Z, Farooqui F, Ibrahim A et al Patients and surfaces: integrated clinical–environmental surveillance of MDR Gram-negative bacteria in critical-care units (Karachi, 2024–2025). Microorganisms 2025; 13: 2762. 10.3390/microorganisms13122762 41471966 PMC12735442

[43] Majigo M, Sukanyi C, Makaria EH et al Bacterial pathogen profile and antimicrobial resistance in neonates with bloodstream infections in Dar es Salaam, Tanzania. Bull Natl Res Cent 2025; 49: 63. 10.1186/s42269-025-01359-7

[44] Yartey SN-A, Kungu F, Asantewaa AA et al Extended spectrum beta-lactamase-producing bacterial clones in West Africa: a systematic review and meta-analysis from a one health perspective. Sci Rep 2025; 15: 29625. 10.1038/s41598-025-10695-7 40796576 PMC12344016

[45] Kigen C, Muraya A, Wachira J et al The first report of the mobile colistin resistance gene, mcr-10.1, in Kenya and a novel mutation in the phoQ gene (S244T) in a colistin-resistant *Enterobacter cloacae* clinical isolate. Microbiol Spectr 2024; 12: e0185523. 10.1128/spectrum.01855-23 38230935 PMC10846102

[46] Liu X, Gong L, Liu E et al Characterization of the disinfectant resistance genes qacE D 1 and cepA in carbapenem-resistant *Klebsiella pneumoniae* isolates. Am J Trop Med Hyg 2024; 110: 136–41. 10.4269/ajtmh.23-0247 38081061 PMC10793004

[47] Eladawy M, Heslop N, Negus D et al Phenotype–genotype discordance in antimicrobial resistance profiles of Gram-negative uropathogens recovered from catheter-associated urinary tract infections in Egypt. J Antimicrob Chemother 2025; 80: 3123–32. 10.1093/jac/dkaf352 40985147 PMC12596052

[48] Baron A, Madhi F, Bidet P et al Epidemiology of aminoglycosides resistance and phenotypic detection of aac(6′)-Ib-cr gene in ESBL-producing Enterobacterales in febrile urinary tract infection in children. J Glob Antimicrob Resist 2025; 44: 135–8. 10.1016/j.jgar.2025.06.008 40543559

[49] Smriti S, Verma G, Pradhan S et al Co-occurrence of genes encoding carbapenem resistance and aminoglycoside resistance in clinical isolates of Enterobacterales. Drug Target Insights 2025; 19: 91–8. 10.33393/dti.2025.3592 41170414 PMC12569621

[50] Logan LK, Coy LR, Pitstick CE et al The role of the plasmid-mediated fluoroquinolone resistance genes as resistance mechanisms in pediatric infections due to Enterobacterales. Front Cell Infect Microbiol 2023; 13: 1249505. 10.3389/fcimb.2023.1249505 37900312 PMC10613066

[51] Sands K, Carvalho MJ, Portal E et al Characterization of antimicrobial-resistant Gram-negative bacteria that cause neonatal sepsis in seven low- and middle-income countries. Nat Microbiol 2021; 6: 512–23. 10.1038/s41564-021-00870-7 33782558 PMC8007471

[52] Peng M, Lin W, Zhou A et al High genetic diversity and different type VI secretion systems in Enterobacter species revealed by comparative genomics analysis. BMC Microbiol 2024; 24: 26. 10.1186/s12866-023-03164-6 38238664 PMC10797944

[53] Lee GY, Song J. Single missense mutations in Vi capsule synthesis genes confer hypervirulence to Salmonella Typhi. Nat Commun 2024; 15: 5258. 10.1038/s41467-024-49590-6 38898034 PMC11187135

[54] Xu L, Wan F, Fu H et al Emergence of colistin resistance gene mcr—10 in Enterobacterales isolates recovered from fecal samples of chickens, slaughterhouse workers, and a nearby resident. Microbiol Spectr 2022; 10: e0041822. 10.1128/spectrum.00418-22 35412362 PMC9045214

[55] Qin J, Wang Z, Xu H et al Incx3 plasmid-mediated spread of blaNDM gene in Enterobacteriaceae among children in China. J Glob Antimicrob Resist 2024; 37: 199–207. 10.1016/j.jgar.2024.03.021 38641225

