# Supplementary material for: Molecular epidemiology and antimicrobial resistance of extended-spectrum beta-lactamases-producing Enterobacter cloacae complex among mothers, neonates, healthcare workers and hospital environments in Tanga, Tanzania
Source: JAC Antimicrob Resist. 2026 Aug 3;8(4):dlag161. doi: 10.1093/jacamr/dlag161 (PMC13430656; doi:10.1093/jacamr/dlag161)
Supplement: dlag161_Supplementary_Data [file dlag161_supplementary_data.zip › Supplementary material 1.docx]

**Library Preparations**

Libraries were prepared using rolling circle amplification to generate DNA nanoballs, followed by combinatorial probe-anchor synthesis (cPAS) sequencing, according to the manufacturer's protocols. ^23^

**Quality control and preprocessing**

Raw short reads were quality filtered using fastp v0.23.4, ^24^ applying sliding-window trimming (window size: 4 bp; quality threshold: Q30), an average read quality threshold of Q30, a minimum read length of 50 bp, and base correction by read overlap.

**Taxonomic classification and genomic features**

Taxonomic classification was performed with GTDB-Tk v2.4.0 ^29^ (database r220). AMR genes were predicted with AMRFinderPlus v4.0.3 ^30^ with the *Enterobacter_cloacae* profile (database version 2024-12-18.1). Plasmids and other mobile genetic elements (MGEs) were analysed using Mob-Suite v3.1.8 (mob_recon). ^31^ Virulence genes were determined with ABRicate v1.0.0 using the VFDB database.

**Phylogenetic analysis**

Phylogenetic tree was generated using tidyverse, ggtree, readxl, openxlsx, janitor, dplyr, and aplot. ^33^
