# Supplementary material for: Molecular epidemiology and antimicrobial resistance of extended-spectrum beta-lactamases-producing Enterobacter cloacae complex among mothers, neonates, healthcare workers and hospital environments in Tanga, Tanzania
Source: JAC Antimicrob Resist. 2026 Aug 3;8(4):dlag161. doi: 10.1093/jacamr/dlag161 (PMC13430656; doi:10.1093/jacamr/dlag161)
Supplement: dlag161_Supplementary_Data [file dlag161_supplementary_data.zip › Supplementary_ Table _S8_ S9.docx]

Supplementary Table S8**:** Further description of mother and neonate samples by presence of ESBL-producing E. cloacae complex

|  | ESBL-ECC positive | Total | p-value |
| --- | --- | --- | --- |
| **Mother samples** |  |  |  |
| N | 11 (1.2%) | 899 (100.0%) |  |
| Age of mother (years) | 27.0 [22.0-33.0] | 27.0 [22.0-32.0] | 0.793 |
| Number of pregnancies (including current) | 3.0 [1.0-4.0] | 2.0 [1.0-3.0] | 0.464 |
| Antibiotic treatment for infection in current pregnancy |  |  |  |
| No | 3 (0.7%) | 423 (47.1%) | 0.107 |
| Yes | 7 (1.5%) | 458 (50.9%) |  |
| Unknown | 1 (5.6%) | 18 (2.0%) |  |
| Any underlying medical condition | 1 (1.9%) | 53 (5.9%) | 0.489 |
| Gestational Diabetes diagnosed during current pregnancy | 0 (0.0%) | 3 (0.3%) | >0.999 |
| Pregnancy Induced Hypertension diagnosed during current pregnancy | 0 (0.0%) | 33 (3.7%) | >0.999 |
| USG performed during this pregnancy | 10 (1.4%) | 707 (78.6%) | 0.474 |
| Foul-smelling amniotic fluid | 0 (0.0%) | 16 (1.8%) | >0.999 |
| Maternal pyrexia > 38.0°C | 0 (0.0%) | 6 (0.7%) | >0.999 |
| **Neonate delivery samples** |  |  |  |
| N | 16 (1.9%) | 826 (100.0%) |  |
| Birth weight (kg) | 3.0 [2.8-3.5] | 3.0 [2.7-3.4] | 0.781 |
| Sex of neonate female | 10 (2.6%) | 380 (46.0%) | 0.211 |
| Caesarean section performed | 7 (2.1%) | 336 (40.7%) | 0.802 |
| Age of mother (years) | 29.0 [23.0-33.5] | 26.0 [22.0-32.0] | 0.352 |
| Number of Pregnancies (including current) | 3.0 [1.0-4.0] | 2.0 [1.0-3.0] | 0.284 |
| Antibiotic treatment for infection in current pregnancy |  |  |  |
| No | 8 (2.1%) | 388 (47.0%) | >0.999 |
| Yes | 8 (1.9%) | 422 (51.1%) |  |
| Unknown | 0 (0.0%) | 15 (1.8%) |  |
| Any underlying medical condition | 1 (2.2%) | 46 (5.6%) | 0.604 |
| Gestational Diabetes diagnosed during current pregnancy | 1 (33.3%) | 3 (0.4%) | 0.058 |
| Pregnancy Induced Hypertension diagnosed during current pregnancy | 1 (3.4%) | 29 (3.5%) | 0.439 |
| USG performed during this pregnancy | 13 (2.0%) | 651 (78.8%) | >0.999 |
| Foul-smelling amniotic fluid | 1 (7.1%) | 14 (1.7%) | 0.243 |
| Maternal pyrexia > 38.0°C | 0 (0.0%) | 5 (0.6%) | >0.999 |
| **Neonate discharge samples** |  |  |  |
| N | 18 (10.2%) | 176 (100.0%) |  |
| Birth weight (kg) | 3.1 [2.7-3.6] | 3.0 [2.7-3.4] | 0.581 |
| Sex of neonate female | 7 (9.0%) | 78 (44.3%) | 0.803 |
| Neonate stayed in hospital more than 48 hours | 18 (10.2%) | 176 (100.0%) | . |
| Caesarean section performed | 17 (12.5%) | 136 (77.3%) | 0.078 |
| Age of mother (years) | 25.5 [22.0-32.0] | 28.0 [22.0-32.0] | 0.660 |
| Number of Pregnancies (including current) | 2.0 [1.0-3.0] | 2.0 [1.0-4.0] | 0.807 |
| Antibiotic treatment for infection in current pregnancy |  |  |  |
| No | 12 (14.0%) | 86 (48.9%) | 0.282 |
| Yes | 6 (7.0%) | 86 (48.9%) |  |
| Unknown | 0 (0.0%) | 4 (2.3%) |  |
| Any underlying medical condition | 2 (14.3%) | 14 (8.0%) | 0.639 |
| Gestational Diabetes diagnosed during current pregnancy |  | 0 (0.0%) |  |
| Pregnancy Induced Hypertension diagnosed during current pregnancy | 0 (0.0%) | 7 (4.0%) | >0.999 |
| USG performed during this pregnancy | 14 (9.7%) | 145 (82.4%) | 0.528 |
| Foul-smelling amniotic fluid | 0 (0.0%) | 3 (1.7%) | >0.999 |
| Maternal pyrexia > 38.0°C | 0 (0.0%) | 1 (0.6%) | >0.999 |

Categorical variable cells are n (%), p-values from Fisher's exact tests

Continuous variable cells are median [IQR], p-values from Kruskal-Wallis rank tests

Supplementary Table S9 **:** Factors potentially associated with colonisation of multi-drug resistant ECC (45 mother and neonate samples)

|  | Multi-drug | Logistic Regression | | | | | |
| --- | --- | --- | --- | --- | --- | --- | --- |
|  | Resistant | Unadjusted | | | Adjusted | | |
|  | n (%) | OR | 95% CI | p-value | OR | 95% CI | p-value |
| N | 29 (64.4%) |  |  |  |  |  |  |
| Hospital |  |  |  |  |  |  |  |
| TRRH | 18 (60.0%) | 1.00 |  |  | 1.00 |  |  |
| KTCH | 11 (73.3%) | 1.83 | 0.47,7.13 | 0.382 | 5.89 | 0.68,50.99 | 0.107 |
| Ward |  |  |  |  |  |  |  |
| Labour | 27 (65.9%) | 1.00 |  |  | 1.00 |  |  |
| Neonatal | 2 (50.0%) | 0.52 | 0.07,4.08 | 0.533 | 0.11 | 0.00,2.47 | 0.164 |
| Age of mother (years) | 29.0 [22.0-34.0] | 1.05 | 0.95,1.15 | 0.337 | 1.36 | 1.05,1.77 | 0.022 |
| Number of Pregnancies (including current) | 3.0 [1.0-4.0] | 1.00 | 0.66,1.51 | 0.996 | 0.27 | 0.08,0.89 | 0.031 |
| Antibiotic treatment for infection in current pregnancy |  |  |  |  |  |  |  |
| No | 14 (60.9%) | 1.00 |  |  | 1.00 |  |  |
| Yes | 14 (66.7%) | 1.29 | 0.37,4.42 | 0.690 | 0.77 | 0.15,3.99 | 0.760 |
| Any underlying medical condition |  |  |  |  |  |  |  |
| No | 26 (63.4%) | 1.00 |  |  | 1.00 |  |  |
| Yes | 3 (75.0%) | 1.73 | 0.16,18.16 | 0.647 | 2.26 | 0.18,29.17 | 0.531 |
| USG performed during this pregnancy |  |  |  |  |  |  |  |
| No | 7 (87.5%) | 1.00 |  |  | 1.00 |  |  |
| Yes | 22 (59.5%) | 0.21 | 0.02,1.88 | 0.163 | 0.22 | 0.02,2.41 | 0.213 |

Categorical variable cells in MDR column are n (%)

Continuous variable cells in MDR column are median [IQR]
