# Supplementary material for: Molecular epidemiology and antimicrobial resistance of extended-spectrum beta-lactamases-producing Enterobacter cloacae complex among mothers, neonates, healthcare workers and hospital environments in Tanga, Tanzania
Source: JAC Antimicrob Resist. 2026 Aug 3;8(4):dlag161. doi: 10.1093/jacamr/dlag161 (PMC13430656; doi:10.1093/jacamr/dlag161)
Supplement: dlag161_Supplementary_Data [file dlag161_supplementary_data.zip › Supplementary_Table_S2_S3_S4.docx]

**Supplementary Table_ S2a: Factors associated with colonisation of multi-drug resistant ECC**

|  | Multi-drug | Logistic Regression | | | | | |
| --- | --- | --- | --- | --- | --- | --- | --- |
|  | Resistant | Unadjusted | | | Adjusted | | |
|  | n (%) | OR | 95% CI | p-value | OR | 95% CI | p-value |
| N | 98 (74.2%) |  |  |  |  |  |  |
| Hospital |  |  |  |  |  |  |  |
| TRRH | 62 (73.8%) | 1.00 |  |  | 1.00 |  |  |
| KTCH | 36 (75.0%) | 1.06 | 0.47,2.40 | 0.880 | 1.06 | 0.46,2.43 | 0.890 |
| Ward |  |  |  |  |  |  |  |
| Labour | 58 (69.9%) | 1.00 |  |  | 1.00 |  |  |
| Neonatal | 40 (81.6%) | 1.92 | 0.81,4.54 | 0.139 | 1.50 | 0.58,3.90 | 0.406 |
| Source of sample |  |  |  |  |  |  |  |
| Mothers & Neonates | 29 (64.4%) | 1.00 |  |  | 1.00 |  |  |
| Environment | 69 (79.3%) | 2.11 | 0.95,4.71 | 0.067 | 1.79 | 0.74,4.34 | 0.199 |

**Supplementary Table S2b. Distribution of β-lactamase and carbapenemase genes among ECC isolates (n = 120)**

| **Gene / Gene Group** | Resistance Type | Isolates (n/N) | Prevalence (%) |
| --- | --- | --- | --- |
| **blaCTX-M (group)** | ESBL | 107/120 | 89.2 |
| **blaCTX-M-15** | ESBL | 105/120 | 87.5 |
| **blaTEM-1** | Broad-spectrum β-lactamase | 73/120 | 60.8 |
| **blaOXA-1** | Broad-spectrum β-lactamase | 59/120 | 49.2 |
| **blaACT (group)** | AmpC β-lactamase | 115/120 | 95.8 |
| **blaACT-15** | AmpC β-lactamase | 30/120 | 25.0 |
| **blaCMH-24** | AmpC β-lactamase | 3/120 | 2.5 |
| **blaCMH-18** | AmpC β-lactamase | 1/120 | 0.8 |
| **blaDHA-1** | AmpC β-lactamase | 1/120 | 0.8 |
| **Carbapenemase genes (overall)** | Carbapenem resistance | 13/120 | 10.8 |
| **blaNDM-1** | Carbapenemase | 7/120 | 5.8 |
| **blaNDM-5** | Carbapenemase | 6/120 | 5.0 |
| **blaOXA-181** | Carbapenemase | 1/120 | 0.8 |

**Supplementary Table S2c. Distribution of other antimicrobial resistance genes among ECC isolates (n = 120)**

| **Resistance Class** | Gene | Isolates (n/N) | Prevalence (%) |
| --- | --- | --- | --- |
| **Fosfomycin** | fosA | 110/120 | 91.7 |
| **Aminoglycosides** | aph(6)-Id / aph(3'')-Ib | 82/120 | 68.3 |
| **Sulfonamides** | sul2 | 83/120 | 69.2 |
| **Sulfonamides** | sul1 | 15/120 | 12.5 |
| **Fluoroquinolones** | oqxA / oqxB | 108/120 | 90.0 |
| **Fluoroquinolones** | qnrB1 | 90/120 | 74.2 |
| **Trimethoprim** | dfrA14 | 68/120 | 56.7 |
| **Trimethoprim** | any dfrA gene | 106/120 | 88.3 |
| **Tetracycline** | tet(A) | 55/120 | 45.8 |
| **Colistin** | mcr-10.1 | 9/120 | 7.5 |
| **Macrolides** | mph(A) | 9/120 | 7.5 |
| **Rifamycin** | arr-3 | 7/120 | 5.8 |
| **Bleomycin** | ble | 12/120 | 10.0 |

**Supplementary Table S3. Localization of resistance genes among ECC isolates**

| **Resistance Gene / Class** | **Plasmid-borne (n)** | **Chromosomal (n)** | **Total Occurrences (n)** |
| --- | --- | --- | --- |
| **β-lactamase genes** |  |  |  |
| blaTEM-1 | 75 | 1 | 76 |
| blaACT | 0 | 93 | 93 |
| blaCMH | 0 | 5 | 5 |
| blaCTX-M-15 | 84 | 25 | 109 |
| blaOXA-1 | 16 | 47 | 63 |
| **Aminoglycoside resistance genes** | 268 | 140 | 408 |
| **Quinolone resistance genes** | 93 | 128 | 221 |
| **Fosfomycin resistance genes** | 0 | 113 | 113 |
| **Phenicol resistance genes** | 18 | 87 | 105 |
| **Tetracycline resistance genes** | 36 | 24 | 60 |
| **Sulfonamide resistance genes** | 170 | 2 | 172 |
| **Trimethoprim resistance genes** | 76 | 42 | 118 |
| **Colistin (mcr-10.1)** | 1 | 8 | 9 |
| **Rifampicin (arr-3)** | 3 | 6 | 9 |
| **Bleomycin (ble)** | 9 | 5 | 14 |
| **Macrolide (mph(A))** | 5 | 6 | 11 |

**Supplementary Table S4. Distribution of ECC sequence types by source (n = 120)**

|  |  |  |  |  |
| --- | --- | --- | --- | --- |
| **ST (n=41)** | **Environmental** | **Neonate** | **Mother** | **Total** |
| 66 | 7 | 2 | 0 | 9 |
| 68 | 2 | 0 | 0 | 2 |
| 84 | 0 | 1 | 2 | 3 |
| 109 | 7 | 3 | 0 | 10 |
| 114 | 2 | 0 | 0 | 2 |
| 120 | 1 | 0 | 0 | 1 |
| 121 | 1 | 0 | 0 | 1 |
| 124 | 1 | 0 | 0 | 1 |
| 127 | 0 | 0 | 1 | 1 |
| 144 | 0 | 1 | 1 | 2 |
| 148 | 0 | 1 | 0 | 1 |
| 171 | 5 | 0 | 0 | 5 |
| 182 | 1 | 0 | 0 | 1 |
| 200 | 2 | 1 | 0 | 3 |
| 270 | 1 | 1 | 0 | 2 |
| 306 | 1 | 1 | 0 | 2 |
| 344 | 1 | 2 | 0 | 3 |
| 346 | 22 | 5 | 3 | 30 |
| 544 | 0 | 1 | 0 | 1 |
| 755 | 2 | 0 | 0 | 2 |
| 916 | 1 | 0 | 0 | 1 |
| 949 | 1 | 0 | 0 | 1 |
| 974 | 1 | 0 | 0 | 1 |
| 1077 | 0 | 1 | 0 | 1 |
| 1241 | 1 | 0 | 0 | 1 |
| 1518 | 0 | 2 | 0 | 2 |
| 1712 | 0 | 1 | 0 | 1 |
| 2093 | 0 | 0 | 1 | 1 |
| 2713 | 5 | 3 | 2 | 10 |
| 3328 | 1 | 0 | 0 | 1 |
| 3369 | 4 | 0 | 0 | 4 |
| 3373 | 1 | 0 | 0 | 1 |
| 3374 | 0 | 1 | 0 | 1 |
| 3375 | 0 | 1 | 0 | 1 |
| 3376 | 0 | 1 | 0 | 1 |
| 3377 | 1 | 0 | 0 | 1 |
| 3378 | 0 | 1 | 0 | 1 |
| 3379 | 4 | 0 | 1 | 5 |
| 3383 | 0 | 1 | 0 | 1 |
| 3385 | 1 | 0 | 0 | 1 |
| 3386 | 1 | 0 | 0 | 1 |
| **Total isolates** | **78** | **31** | **11** | **120** |
|  |  |  |  |  |
| **Summary** |  |  |  |  |
| **Category** | **Count** |  |  |  |
| Total isolates | 120 |  |  |  |
| Distinct STs | **41** |  |  |  |
| **STs shared between ≥2 sources** | **11** |  |  |  |
| STs unique to a single source | 30 |  |  |  |
