# Supplementary material for: Molecular epidemiology and antimicrobial resistance of extended-spectrum beta-lactamases-producing Enterobacter cloacae complex among mothers, neonates, healthcare workers and hospital environments in Tanga, Tanzania
Source: JAC Antimicrob Resist. 2026 Aug 3;8(4):dlag161. doi: 10.1093/jacamr/dlag161 (PMC13430656; doi:10.1093/jacamr/dlag161)
Supplement: dlag161_Supplementary_Data [file dlag161_supplementary_data.zip › Supplementary_Table_S6_S7.docx]

**Supplementary Table S6. Distribution of resistant genes among the ECC isolates**

| **Virulence Category** | **Gene / Operon** | **Function** | **Isolates with Gene (n/N)** | **Prevalence (%)** |
| --- | --- | --- | --- | --- |
| **Adhesion & Biofilm Formation** | ompA | Outer membrane protein; adhesion and immune evasion | 120/120 | 100 |
|  | csgG | Curli assembly; biofilm formation | 67/120 | 55.8 |
|  | lpfABCE | Long polar fimbriae; adhesion | 3/120 | 2.5 |
| **Iron Acquisition (Siderophore Systems)** | iroBCDEN | Salmochelin siderophore system | 51/120 | 42.5 |
|  | entA | Enterobactin synthesis | 3/120 | 2.5 |
|  | entB | Enterobactin synthesis | 14/120 | 11.7 |
|  | fepD | Ferric enterobactin transport | 7/120 | 5.8 |
|  | fyuA | Yersiniabactin receptor | 3/120 | 2.5 |
|  | irp | Yersiniabactin biosynthesis | 3/120 | 2.5 |
|  | ybtAEPQSTUX cluster | Yersiniabactin siderophore system | 3/120 | 2.5 |
| **Capsule & Efflux-Associated Virulence** | tviBCDE | Capsular polysaccharide biosynthesis | 8/120 | 6.6 |
|  | vexABCDE | Efflux transporter associated with virulence | 8/120 | 6.6 |
| **Toxin Genes** | astA | Heat-stable enterotoxin | 1/120 | 0.8 |

**Supplementary Table S7. Distribution of plasmid incompatibility type among the ECC isolates**

| **Incompatibility (Inc) Type** | **Isolates with Inc Type (n/N)** | **Prevalence (%)** |
| --- | --- | --- |
| IncR | 45/120 | 37.5 |
| IncFIB(K) | 33/120 | 27.5 |
| Col440I | 33/120 | 27.5 |
| IncFIA(HI1) | 30/120 | 25.0 |
| IncFII(Yp) | 25/120 | 20.8 |
| IncHI2 | 17/120 | 14.2 |
| IncHI2A | 17/120 | 14.2 |
| IncX3 | 8/120 | 6.6 |
| IncFIB(pECLA) | 7/120 | 5.8 |
| IncFII(pECLA) | 6/120 | 5.0 |
| IncY | 5/120 | 4.2 |
| IncFIB(pB171) | 3/120 | 2.5 |
| RepA | 2/120 | 1.7 |
| IncN | 2/120 | 1.7 |
| IncN3 | 1/120 | 0.8 |
| IncFIB(pHCM2) | 1/120 | 0.8 |
| IncX3(pEC14) | 1/120 | 0.8 |
